# Non-Classic Disorder of Adrenal Steroidogenesis and Clinical Dilemmas in 21-Hydroxylase Deficiency Combined with Backdoor Androgen Pathway. Mini-Review and Case Report

**DOI:** 10.3390/ijms21134622

**Published:** 2020-06-29

**Authors:** Marta Sumińska, Klaudia Bogusz-Górna, Dominika Wegner, Marta Fichna

**Affiliations:** 1 Department of Pediatric Diabetes and Obesity, Poznan University of Medical Sciences, 60-527 Poznan, Poland; kl.bogusz@gmail.com (K.B.-G.); dominika.wegner@op.pl (D.W.); 2 Department of Endocrinology, Metabolism and Internal Medicine, Poznan University of Medical Sciences, 60-653 Poznan, Poland; mfichna@ump.edu.pl

**Keywords:** non-classic congenital adrenal hyperplasia, 21-hydroxylase deficiency, backdoor androgen pathway, 16α-hydroxydehydroepiandrosterone (16α-OHDHEA), pregnanetriolone (PTN)

## Abstract

Congenital adrenal hyperplasia (CAH) is the most common cause of primary adrenal insufficiency in children and adolescents. It comprises several clinical entities associated with mutations in genes, encoding enzymes involved in cortisol biosynthesis. The mutations lead to considerable (non-classic form) to almost complete (classic form) inhibition of enzymatic activity, reflected by different phenotypes and relevant biochemical alterations. Up to 95% cases of CAH are due to mutations in *CYP21A2* gene and subsequent 21α-hydroxylase deficiency, characterized by impaired cortisol synthesis and adrenal androgen excess. In the past two decades an alternative (“backdoor”) pathway of androgens’ synthesis in which 5α-androstanediol, a precursor of the 5α-dihydrotestosterone, is produced from 17α-hydroxyprogesterone, with intermediate products 3α,5α-17OHP and androsterone, in the sequence and with roundabout of testosterone as an intermediate, was reported in some studies. This pathway is not always considered in the clinical assessment of patients with hyperandrogenism. The article describes the case of a 17-year-old female patient with menstrual disorders and androgenization (persistent acne, advanced hirsutism). Her serum dehydroepiandrosterone sulfate and testosterone were only slightly elevated, along with particularly high values for 5α-dihydrotestosterone. In 24 h urine collection, an increased excretion of 16α-OHDHEA—a dehydroepiandrosterone metabolite—and pregnanetriolone—a 17α-hydroxyprogesterone metabolite—were observed. The investigations that we undertook provided evidence that the girl suffered from non-classic 21α-hydroxylase deficiency with consequent enhancement of the androgen “backdoor” pathway in adrenals, peripheral tissues or both, using adrenal origin precursors. The paper presents diagnostic dilemmas and strategies to differentiate between various reasons for female hyperandrogenism, especially in childhood and adolescence.

## 1. Introduction

### 1.1. Relationship between Adrenal Cortex Structure and Function

The adrenal glands have a complex structure and function. They consist of an outer adrenal cortex with three distinguishable zones and an inner adrenal medulla, characterized by centripetal blood flow. The cortex is the place of complex and multistage processes of steroidogenesis, which initially proceeds along a common pathway and then, depending on the zone, leads to production of mineralocorticoids (zona glomerulosa), glucocorticoids (zona fasciculata) and adrenal androgens (zona reticularis) synthesis. The activity of zona glomerulosa enzymes is modulated by the renin-angiotensin-aldosterone system, while two other main pathways remain under control of the hypothalamic-pituitary-adrenal (HPA) axis. Adrenocorticotropic hormone (ACTH) stimulates the secretion of cortisol (z. fasciculata) and adrenal androgens (z. reticularis) [1]. However, the negative feedback response relies uniquely upon suppressive effect of circulating cortisol on corticotropin-releasing hormone (CRH) and ACTH release, whereas adrenal androgens cannot exert similar influences on the HPA axis [2]. Moreover, the vascular system in the adrenal gland enables it to supply steroidogenic cells in zona reticularis with high local concentrations of precursor steroids (e.g., 17α-hydroxyprogesterone, 17OHP) from zona fasciculata, and this influx is further enhanced if there are enzymatic deficiencies in the glucocorticoid pathway [3]. This process illustrates the ultra-short endocrine (blood transport is engaged), paracrine (both zones are neighboring tissues) or even intracrine mechanisms (precursor steroids from zona fasciculata are processed to androgens inside the cells of zona reticularis) implicated in adrenal steroidogenesis.

### 1.2. Congenital Adrenal Hyperplasia and Androgen Biosynthesis

Congenital adrenal hyperplasia (CAH) is the most common cause of primary adrenal insufficiency in children and adolescents [1]. It comprises several clinical entities associated with mutations in distinct genes, which encode enzymes involved in cortisol biosynthesis. The ensuing enzymatic defects, inherited in an autosomal recessive manner, lead to considerable reduction or almost complete inhibition of their activity. The result of these disorders is cortisol deficiency coexisting with excess or deficiency of mineralocorticoids and adrenal androgens. Clinical consequences of these mutations are reflected by different phenotypes and relevant changes in the laboratory tests, depending on specific enzyme affected and the degree of its dysfunction (almost total deficit results in classic disease form, while partially limited enzymatic activity leads to non-classic forms). Up to 95% of CAH cases are due to mutations in *CYP21A2* gene and subsequent deficit of 21α-hydroxylase [4,5]. Less common reasons comprise mutations of *CYP11B1* which result in 11β-hydroxylase deficiency (3–4% of CAH cases) and other (1–2%) very rare enzymatic blocks [5,6].

Since the core problem in classic CAH is cortisol shortage, the leading aim of therapy is its substitution as well as suppression of the hypothalamus (CRH) and pituitary gland (ACTH) with exogenous glucocorticoid in order to achieve inhibition of excess androgen production [7,8]. The first-choice drug is usually hydrocortisone, identical to natural cortisol, at a dose of 10–18 mg/m^2^ of the body surface divided into three (four) doses per day [9,10]. However, in non-classic CAH forms, where levels of endogenous cortisol remain normal and values of other metabolites are only moderately elevated, the inclusion of suppressive treatment to bring the elevated CRH and ACTH levels down may generate adverse effects due to supra-physiological dosing of glucocorticoids. On the other hand, each unblocked ACTH burst will result in transient excess of androgen secretion. Therefore, proper and well-balanced treatment is of critical importance in this condition, and this is only possible with an accurate diagnosis. In non-classic, subtle disorders of adrenal steroidogenesis diagnostic procedures are often bothersome and require meticulous analyses. Even if many of these disorders start early in childhood, their clinical manifestation is very mild, but usually exacerbates later on, e.g., at puberty, leading to overt symptoms in adolescent girls and adult women, who may present with disturbed ovarian function. 

**Figure 1:**
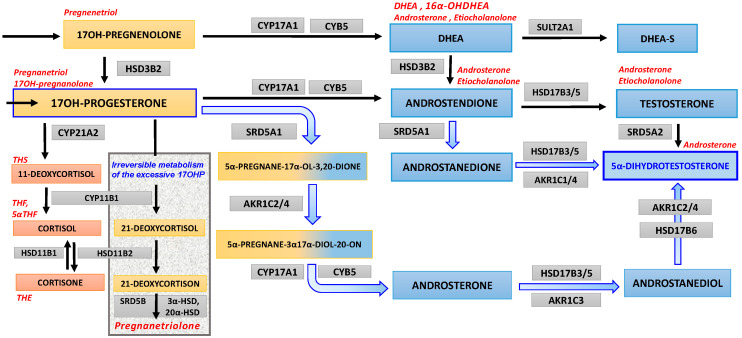
Androgen “backdoor” steroidogenesis pathway. The orange boxes bear the names of the steroid precursors at early stages of steroidogenesis and for 17OHP and its derivatives; reddish boxes for the cortisol pathway and its metabolite; blue areas (and mixed orange/blue) are background for androgens and their precursors; and finally gray boxes bear names of enzymes, which are involved in consecutive steps of steroidogenesis. The bluish arrows depict steps of the androgen backdoor pathway. The left side of the figure presents a few steps of steroidogenesis leading to cortisol synthesis (steroidogenesis beginning from cholesterol towards mineralocorticoid pathway is omitted), which are adjacent the rectangle area displaying the irreversible pathway from 17OH-progesterone to pregnanetriolone, a marker of adrenal origin of 17OHP. The upper row (from 17OH-pregnenolone) presents canonical steroidogenic steps to dehydroepiandrosterone (and its sulfate), which initiate progression of so-called classic androgen pathways toward testosterone and thereafter, to 5α-dihydrotestosterone. The main substrate appears to be 17OH-progesterone, which fuels (broad bluish arrows) the ”androgen backdoor pathway" and eventually reaches 5α-dihydrotestosterone with omission of testosterone as an intermediate step. The abbreviations of the enzymes involved in each step are displayed in gray boxes: AKR1C1/4 aldo-keto reductase 1C1/1C4 (3αHSD), AKR1C2/4 aldo-keto reductase 1C2/1C4 (3αHSD), AKR1C3 aldo-keto reductase 1C3 (17β-hydroxysteroid dehydrogenase type 5), CYB5 cytochrome b5, CYP11B1 cytochrome P450 11β-hydroxylase, CYP17A1 cytochrome P450 17α-hydroxylase/17,20-lyase, CYP21A2 cytochrome P450 21α-hydroxylase, HSD11B1 11β-hydroxysteroid dehydrogenase type 1 (mainly reductase 11BHSD), HSD11B2 11β-hydroxysteroid dehydrogenase type 2 (mainly oxidase 11BHSD), HSD3B2 3b-hydroxysteroid dehydrogenase type 2, HSD17B3/5 17β-hydroxysteroid dehydrogenase type 3/type 5, HSD17B6 17β-hydroxysteroid dehydrogenase type 6, 3αHSD 3α-hydroxysteroid dehydrogenase, 20αHSD 20α-hydroxysteroid dehydrogenase, SRD5A1 steroid 5α-reductase type 1, SRD5A2 steroid 5α-reductase type 2, SRD5B steroid 5β-reductase (AKR1D1 5b-reductase), SULT2A1 sulfotransferase 2A1 (DHEA sulfotransferase). Steroid metabolites usually estimated in urines are indicated in red letters close to their steroid precursors.

In the past two decades an alternative (“backdoor”) pathway of androgens’ syntheses in which 5α-androstanediol, precursor of the 5α-dihydrotestosterone (DHT), is produced from 17OHP, with intermediate products 3α,5α-17OHP and androsterone, in the sequence and with roundabout of testosterone as an intermediate product, was reported in some studies (Figure 1) [11,12,13]. Such a pathway is not always taken into account in the clinical assessment of patients with hyperandrogenism, hence this may be a source of diagnostic pitfalls and confusion.

## 2. Case Report

A 17-year-old girl (menarche at 12 y) presented with oligomenorrhea (menstrual cycles of 40–75 days) and hyperandrogenism observed for the last few years, since puberty. She suffered from persistent, severe acne, oily skin and hirsutism (modified Ferriman–Gallway score 11). Her weight was 57 kg, height 168 cm and body mass index (BMI) 20.2 kg/m^2^. 

The patient was admitted to the Children’s Hospital at Poznan University of Medical Sciences. Written consent for routine diagnostic and treatment procedures is always taken on admission, together with consent for the use of all diagnostic or treatment results for scientific analysis and anonymous publication. Informed consent was obtained from the patient, aged 17 years old and from her legal representative, i.e., her mother. The approval of the Ethics Committee is not required for case reports as long as there is no experimental or out of label procedures applied. In the described case only routine procedures were performed and the value of our study relies upon meticulous analysis of all collected data. 

Five months earlier she had been admitted into a gynecological ward where the diagnostic tests revealed slightly elevated levels of prolactin, 27.24 ng/mL (reference range: 4.79–23.30), and dehydroepiandrosterone sulfate (DHEA-S), 13.44 µmol/L (reference range: 1.77–9.99). Total testosterone (T) was 2.60 nmol/L (reference range: 0.2–2.80) and sex hormone binding globulin (SHBG), 103.1 nmol/L (reference range: 26.1–110.0). Free androgen index (FAI) was 2.5, and bioavailable testosterone was 0.50 nmol/L, i.e., 19.0%. In an abdominal ultrasound, the uterus size was estimated as 38 × 25 mm, endometrium 6 mm, while ovaries were not described at all. According to the patient’s statement, gynecological examination was not performed at that time. In an oral glucose tolerance test (OGTT) the fasting glucose was 106 mg/dL and in the 120th minute 145 mg/dL; while fasting insulin 14 IU/mL and 73 IU/mL at 120 min post glucose load. Based on these results, insulin resistance was diagnosed (homeostatic model assessment of insulin resistance, HOMA-IR 3.66) with borderline glycemia for impaired tolerance. Treatment with metformin was recommended; however, the patient has never started this therapy.

A few months later her hormonal diagnostic was extended in the pediatric endocrinology ward. The patient supplied her 24 h urine collection completed in the morning of admission (4th/5th day of follicular phase), which was sent for steroid profile evaluation. Thyroid function tests, including thyroid-stimulating hormone (TSH), free thyroxine (fT4) and free triiodothyronine (fT3) were all within the reference range. Other hormonal tests performed on the 6th day of the menstrual cycle revealed serum DHEA-S 11.39 µmol/L, i.e., close to the upper limit of normal, the luteinizing hormone/follicle stimulating hormone (LH/FSH) ratio 9.8/6.3 mIU/mL, i.e., only slightly shifted towards LH, with the estradiol (E2) level 38 pg/mL, i.e., at the lower limit of the reference range. Morning plasma ACTH was 84.9 pg/mL (reference range up to 60 pg/mL) (Table 1). 

The OGTT was repeated with simultaneous estimations of frequently sampled glycemia, serum insulin and C-peptide levels. Its results were within the range of reference (HOMA-IR 1.58) and excluded glycemic-insulin disturbances in the patient (Table 2). 

Standard adrenocortical stimulation (on 7th day of cycle) with 250 μg intravenous Synacthen (an ACTH_1–24_ analogue) revealed normal secretory reaction of cortisol, 17OHP and androstenedione (ANDR) (Table 3). 

The results of steroid metabolites estimation in 24 h urine collection demonstrated an increased amount of 16α-OHDHEA (610.2 µg/24 h)—a dehydroepiandrosterone metabolite—and a marked increase in excretion of pregnanetriolone (PTN, 142.5 µg/24 h) (Table 4). 

In conclusion to their analysis, the steroid laboratory suggested excluding CAH in this patient. However, according to the clinical endocrinologist, the selective imbalance of excreted steroid metabolites was probably due to a mild restriction of 21α-hydroxylase (elevated PTN was suggestive of excess 17OHP). The contribution of the alternative pathway leading to enhanced production of DHT, with omission of T, was also suspected because of the discrepancy between severe skin symptoms and formerly reported normal serum testosterone level. 

In the next step, a 3-day dexamethasone (0.5 mg orally every 6 h) suppression test was carried out. Its results (Table 5) revealed initially elevated and then lowered serum values of DHEA-S (16.40 then 4.52 µmol/L), T (3.84 then 2.16 nmol/L) and ANDR (2.59 then 1.78 ng/mL). In the same series, a significantly elevated concentration of DHT (578 pg/mL) was observed and thereafter, upon dexamethasone, it returned to the reference range (337 pg/mL). 

The abdominal ultrasound was also performed—no focal changes were found in the adrenal glands; both ovaries (LO: 33 × 24 × 21 mm, RO: 35 × 17 × 16 mm) were described as having normal solid/follicular architecture and the diameter of the largest follicle in the RO was 11 mm. We have revisited FAI again (for SHBG, 105.0 nmol/L and T, 3.84 nmol/L) and the result was 3.66, while T bioavailability 0.72 nmol/L, i.e., 18.8%. Hence, compared to previous estimations, FAI was slightly higher, however still within normal range.

Summarizing the patient’s medical history and her current clinical and biochemical results, we concluded that there were no convincing arguments to establish the diagnosis of polycystic ovary syndrome (PCOS), especially because adrenal source of hyperandrogenism was beyond doubt. The patient was discharged with the diagnosis of mild late-onset 21α-hydroxylase deficiency (steroid metabolites in urine) and hyperandrogenism due to enhanced “backdoor” androgen pathway. Furthermore, hormonal evaluation suggests a tendency for episodic stress-like reactions, manifested with higher ACTH and baseline cortisol concentrations close to the upper reference limit. However, 24 h urine cortisol excretion remained normal.

Our patient did not fulfill all standard criteria for PCOS diagnosis in adolescents [14,15,16]. She presented irregular menstruation, clinical hyperandrogenism; however, biochemical indices of testosterone excess, as well as insulin resistance were not permanent or significant, and ovaries did not present morphology typical for PCOS. Moreover, the patient’s BMI was normal and stable. Nevertheless, an early stage of PCOS could not be totally excluded in the future besides the confirmed DHT excess of adrenal origin. Therefore, continued care of an endocrinologist-gynecologist was proposed, with the suggestion to start spironolactone treatment, combined with a two-component contraceptive pills comprising drospirenone for their antiandrogenic effects. A healthy lifestyle (diet, physical activity) was also advised as an essential method to avoid progression towards insulin resistance.

## 3. Laboratory Methods

The patient’s BMI was based upon standard anthropometric measurements and calculated as weight (kg)/height (m) squared. Insulin resistance (IR) was diagnosed according to the homeostasis model assessment for IR index (HOMA-IR), and the value was calculated using the following formula: (fasting plasma glucose (mg/dL)/18) × fasting serum insulin (µU/mL)/22.5. We accepted HOMA-IR <2.5 as proper score, between 2.5 and 4.0 as a borderline gray area and above 4.0 as evident IR [17,18].

All biochemical measurements were performed in laboratories of the university reference hospitals. In the gynecological ward insulin, prolactin and SHBG were measured by specific electro-chemiluminescence assays (automated Elecsys 2010 immunoanalyzer, Roche Diagnostics GmbH). The same method, with the use of Cobas 6000 equipment and Roche reagents, was applied to measure total testosterone level. Free androgen index (FAI) was calculated as serum testosterone (nmol/L) × 100/SHBG (nmol/L) ratio. The cut off value for FAI was 6.1 [19]. The chemiluminescence method was used to determine serum level of DHEA-S with the use of Immulite equipment and Siemens reagents. In the pediatric endocrinology ward, serum levels of DHEA-S, T, ANDR, 17OHP and C-peptide were evaluated by specific radioimmunoassay kits (automated Wallac Wizard 1470 Automatic Gamma Counter, Perkin Elmer). Insulin, cortisol, LH, FSH and E2 were measured by chemiluminescence method (Alinity i, Abbott). The standard colorimetric method was used for determination of glucose level (Clinical Chemistry Analyzer AU680, Beckman Coulter). Urinary profile of steroid metabolites was investigated in the reference laboratory (Laboratory of Steroid Hormones, The Children’s Memorial Health Institute, Warsaw) by gas chromatography and mass spectrometry (GC-MS). Quantitative determination of serum DHT level was performed using an ELISA (enzyme-linked immunosorbent assay) kit (Demeditec Diagnostics GmbH, Kiel, Germany) and the absorbance was analyzed on Universal Microplate Reader (Elx800, BioTek Instruments, VT, USA).

## 4. Discussion

### 4.1. Preliminary Suspicion of PCOS

Initially, the studied adolescent with clinical symptoms of hyperandrogenism (severe acne, hirsutism, irregular menstruation) had been consulted by gynecologists. Based on their workup, insulin resistance was assumed and treatment with metformin was recommended, as the routine procedure in polycystic ovary syndrome. However, no documentation of characteristic ovaries’ structure was available. Furthermore, glycemia and insulinemia in the course of OGTT were close to the upper borderline and HOMA-IR remained in the gray zone (a few months later all these measurements were clearly normal). Of note, total T and FAI were not elevated in contrast to evident clinical symptoms of hyperandrogenism. Serum DHEA-S and prolactin concentrations were only slightly above the upper reference limit. Altogether, PCOS has never been fully confirmed and eventually this suspicion was excluded a few months later, in the course of extended clinical investigation by pediatric endocrinologists.

### 4.2. Early Diagnostics of CAH May Miss Its Non-Classic Forms 

The severity and clinical progression of CAH associated with 21α-hydroxylase deficiency depends on the degree of the enzyme deficit. Traditionally, four forms of CAH are distinguished: classic form with or without loss of salt (salt-wasting or simple virilizing, respectively), non-classic form (late-onset form) and cryptogenic form (asymptomatic) [5,8]. In Poland a nationwide screening program for CAH, which covers the entire neonatal population, was launched in 2017 [20]. In the first stage of screening, on the 3rd or 4th day of life, serum level of 17αOH-progesterone is determined. The reference range depends upon the week of pregnancy (hbd) at birth and on the day of life at blood collection. In the second stage of screening the steroid profile focused on 17αOH-progesterone, cortisol, 21-deoxycortisol, 11-deoxycortisol and androstenedione levels is determined in this same blood sample using liquid chromatography tandem mass spectrometry (LC/MS/MS). Still, this procedure does not provide unequivocal information about the risk of late-onset non-classic forms of CAH in the future. In some countries (Spain) there are attempts to also use pregnanetriolone estimation in urine samples collected on the 3rd day of life, using a piece of sorbent paper included in the screening kit to distinguish steroid profile characteristics for preterm infants from those with genuine CAH [21].

### 4.3. Differential between Non-Classic CAH and PCOS

Both PCOS and non-classic CAH are diseases manifesting in hyperandrogenism, which require careful differential diagnosis in adolescent girls and adult women [22]. In the present case, the first diagnostic steps routinely gravitated towards PCOS although this supposition was not fully supported by biochemical and ultrasonography (USG) studies. Subsequent investigations definitively excluded insulin resistance, did not detect changes in ovarian structure and hence focused on adrenal function. Serum T and DHEA-S fluctuated slightly at the upper limit of the reference range with maintained normal FAI and testosterone bioavailability. On the other hand, basal and Synacthen-stimulated cortisol, 17OHP and ANDR levels were not suggestive of late-onset congenital adrenal hyperplasia. Since CAH seemed doubtful, it was necessary to find an alternative source of androgen activity besides testosterone. DHT was the next potential candidate as a likely causative factor for skin symptoms [23,24]. Therefore, serum T and DHT were measured before and after 3-day dexamethasone suppression test. It appeared that basal DHT was almost twice the upper limit of the reference range while testosterone was only slightly elevated before the test. After ACTH suppression with dexamethasone, both androgens revealed significant decrease, which confirmed their predominantly adrenal origin. Moreover, in case of our patient, urinary steroid metabolites did not present a profile typical for polycystic ovary syndrome, characterized by elevated dehydroepiandrosterone, androstenediol and pregnenetriol [25]. As a matter of fact, the current case report is among just a few papers suggesting the relationship between diminished 21-hydroxylase activity and induction of the androgen backdoor pathway. Kamrath et al. [26] reported a retrospective analysis of steroid metabolites and their ratios in urine samples from a number of patients with 21-hydroxylase deficiency and found that androgen backdoor pathway contributed to hyperandrogenism. However, the studied cohort was comprised mainly of prepubertal individuals diagnosed with CAH in early infancy, therefore suffering from a more severe enzyme deficiency. Nonetheless, the authors hypothesized that this pathway might explain divergences between clinical degree of virilization and serum androgen levels, sometimes encountered in endocrine practice. Endocrinologists frequently meet adolescent girls or young women who are suffering with hirsutism, severe acne or both, while their diagnosis of non-classic CAH or PCOS is not fully confirmed [27]. 

### 4.4. Subtle Alterations in Urine Steroid Profile

At that stage, the major task appeared to be working out the mechanism of DHT generation, because it was not clear how to explain the discrepancy between changes in serum concentrations of two active androgens. The canonical androgen steroidogenesis proceeds from DHEA through androstenedione toward testosterone and finally, by way of 5α-reduction, may lead to DHT, which is the most potent and irreversible end-stage metabolite [28]. In the discussed case, there were only slightly elevated serum DHEA-S, fluctuating T and normal ANDR, all not relevant for expected enhanced activity of this pathway, capable of inducing clinically serious hyperandrogenism. In the urinary steroid profile, only two metabolites were excreted at increased rate, 16α-OHDHEA and PTN, while the remaining steroids presented normal excretion rates (Table 4). The 16α-OHDHEA was the only elevated DHEA-S metabolite, while four other DHEA-S derivatives (DHEA, 5-AND, An-3-ol, 5-PT) maintained normal 24 h excretion rate. Synthesis of 16α-OHDHEA typically rises in the course of pregnancy as this DHEA metabolite derived from the placenta serves as a substrate for production of estriol. However, our patient was not pregnant at the time of the study. In the presented female adolescent, the selective increase of urinary 16α-OHDHEA seems consequent to borderline elevation of circulating serum DHEA, a substrate for hepatic 16α-hydroxylase. Actually, this constellation might be due to the postulated mild limitation of 21α-hydroxylase activity, and in line with the observed increase in pregnanetriolone, the uniquely elevated metabolite of 17αOH-progesterone. The amounts and proportions of particular steroid metabolites in serum and urines may be dependent upon concentrations of their precursors and local enzymatic capacity in various tissues responsible for steroid metabolism (adrenals, liver, adipose tissue, etc.). Furthermore, the excretion of PTN was three-fold higher than the reference range, while the other four steroids derived from 17OHP (17-OHPN (5beta), 17-OHPN (5alpha), PT, PD) were excreted at a normal rate. We found it intriguing that only single metabolites of DHEA and 17OHP were elevated in 24 h urine collection of the investigated case. The urine excretion of androsterone and etiocholanolone remained normal in the presented case despite the presumed androsterone role as main intermediate in the backdoor pathway while etiocholanolone is associated rather with the classic pathway toward DHT. Former analyses revealed that later diagnosis of CAH, i.e., presumably milder 21-hydroxylase deficiency, was associated with lower androstenedione to etiocholanolone ratios [26]. However, data from pre- and post-pubertal subjects were not analyzed separately, hence we cannot compare these results with findings from our patient. Furthermore, there is an alternative androgenic way from androstenedione via androstanedione to DHT with omission of androsterone and testosterone as well (Figure 1), which needs to be considered. This pathway employs 5α-reductase type 1 (SRD5A1) and thereafter aldo-keto-reductases 1/4 (AKR1C1/4) with 17β-hydroxysteroid dehydrogenases 3/5 (HSD17B3/5) and might explain lack of elevated androsterone and etiocholanolone. Cortisol and cortisone as well as their metabolite ratios were also normal. It indicated, that there was no imbalance in activity of HSD11B1 and HSD11B2 (Figure 1) which could influence HPA axis and increased androgen production or could interfere with androgen metabolism. Therefore, eventually we had to solve the puzzle of severe clinical symptoms accompanied by only mild to moderate alterations of serum and urinary steroids.

### 4.5. 17OHP Excess as Substrate for “Backdoor” Androgen Synthesis 

Increased secretion of intermediate steroids such as 17OHP and DHEA may suggest 21α-hydroxylase deficiency with maintained normal efficiency of 11β-hydroxylase, although a moderate degree of biochemical disturbance suggests only mild enzymatic block, i.e., non-classic form of CAH. However, an impaired 21α-hydroxylase activity, especially under stressful conditions, results in elevated 17OHP, a substrate for 11β-hydroxylase type 1, which converts it into 21-deoxycortsol, a compound which in turn is metabolized by 11β-hydroxysteroid dehydrogenase type 2 (HSD11B2) into 21-deoxycortisone. Under the triple influence of 5β-reductase, 3α-hydroxysteroid dehydrogenase and 20α-hydroxysteroid dehydrogenase, 21-deoxycortisone is further transformed into PTN, the final urinary metabolite (Figure 1) [12,26,29]. One should bear in mind that PTN or its precursors cannot serve as substrates for androgen production because their generation proceeds via the 5β-reduction pathway and not through 5α-reduction. Therefore, PTN is a marker of elevated 17OHP of adrenal origin only. On the other hand excess 17OHP can still supply 5α-reduction shift toward the “backdoor” androgen formation [11,12,30]. We rather presumed the “backdoor” than the canonical pathway of androgen biosynthesis, because serum T oscillated within normal to high normal levels, while DHT was clearly pathologically elevated although remained reactive to adrenal suppression with dexamethasone. The androgen “backdoor” pathway relies on omission of testosterone on the way leading to DHT hence it seems highly probable that we had such a case to do with here [31]. The accumulation of 17OHP seems critical for both disorders—CAH and enhanced androgen backdoor pathway—as a result of 21-hydroxylase deficiency and as a substrate for the backdoor pathway, respectively. Even a mild/moderate increase of 17OHP may be sufficient to set in motion this steroidogenic route, leading to excess DHT via androsterone. In CAH, this pathway could be parallel or even more potent, than the classic one, leading via androstenedione. On the other hand the androgen backdoor pathway may use some intermediate substrates from adrenals and finish synthesis of DHT locally in different tissues. This was observed in fetal life [30], in newborns [32] and in infants during mini-puberty [33] as well as in some pathologies like prostate cancer [34].

### 4.6. Information from Steroid Metabolites’ Ratios

Taken together, limitation of 21α-hydroxylase activity induces an androgen “backdoor” pathway supplying it with the main substrate, 17OHP [25,29]. In the presented case, there was a considerable increase in the urine metabolites of 17OHP (PTN 142.5 μg/24 h; reference range 10–50 μg/24 h) and DHEA (16α-OHDHEA 610.2 μg/24 h; reference range 50–490 μg/24 h). Furthermore, as recommended by Kamrath et al., we performed calculations of steroid urine metabolite ratios to clarify the previous biochemical findings [26]. In our patient, the ratio of pregnanetriolone/cortisol metabolites (i.e., THF + aTHF + THE) of 0.045 was above the reference range, which supports diminished 21OH-lase activity. In accordance, borderline values were found for two other ratios characteristic for 21α-hydroxylase deficiency: pregnanetriol/cortisol metabolites (0.121) and 17αOH-pregnanolone/cortisol metabolites (0.039). Moreover, a substantially elevated value was observed for androsterone + etiocholanolone/cortisol metabolites (1.284), a feature which is postulated typical for diminished activity of 17βOH-steroid dehydrogenase type 3. This enzymatic deficiency impairs transformation of ANDR to T, and can also corroborate the shift of androgen formation from canonical to “backdoor” androgen pathway through 5α-androstanedione as an intermediate toward DHT (the alternative Δ-5 pathway), by androsterone toward DHT (the “backdoor” pathway) or both. Our patient was an almost adult adolescent girl who presented the ratio of androsterone and etiocholanolone to tetrahydro-derivatives of cortisol and cortisone in urine, which may suggest diminished activity of HSD17B3 [25,35]. The meaning of such a ratio is difficult to explain, while the enzyme is involved in androgen production in different organs, e.g., gonads and peripheral tissues but not especially in adrenals.

### 4.7. Study Limitation—Lack of 11-Oxygenated-19-Carbon Androgens Evaluation

In our patient, the adrenal origin of DHT seemed evident and steroid metabolites in 24 h urine collection indicated increased adrenal 17OHP as a substrate for “backdoor” androgen pathway. Nevertheless, we realize that not all possible tests were done. We did not analyze the full spectrum of androgen steroids, which could influence the clinical status of our patient. According to recent reports, not only T and DHT are considered active androgens, but also 11-oxygenated-19-carbon (11oxC19) adrenal-derived steroids such as 11β-hydroxyandrostenedione (11-OHA4) and 11-ketoandrostenedione (11-KA4), 11β-hydroxytestosterone (11-OHT) and 11-ketotestosterone (11-KT), or both may play a role and should be evaluated [36,37,38,39]. In this pathway 11β-hydroxylase type 1 (CYP11B1) utilizing ANDR and T as substrates, produces 11-OHA4 and 11-OHT, respectively. Both of them are released into circulation and then can be activated in peripheral tissues by conversion with 11β-hydroxysteroid dehydrogenase type 2 (HSD11B2) into their 11-keto forms [40]. In our patient, we were not able to directly assess the concentration of 11oxC19 steroids in the blood, but the correct values of their derivatives (11keto- as well as 11hydroxy- metabolites of androsterone and etiocholanolone) in daily urine collection seemed to rule out their excess. The activity of CYP11B1, HSD11B2 and HSD11B1 is of particular interest, as these enzymes used to be considered as engaged uniquely in glucocorticoid synthesis or metabolism, while it appears that they may be implicated in the androgen pathways too. Serum and local 11-oxygenated C19 steroids can be elevated in disorders of adrenal steroidogenesis as well as in the polycystic ovary syndrome, and in some instances both sources of their excess coexist [37,41].

### 4.8. Serum Levels vs. Paracrine and Intracrine Impact on Symptoms

It is not uncommon that endocrinologists encounter trouble with the interpretation of serum androgens and urinary steroid profiles with respect to a patient’s symptoms. We rarely can base interpretations on Aristotle’s syllogism, because clinical effects usually depend upon several causative factors. The symptoms observed in our patient were due to the local action of androgens, e.g., testosterone and chiefly DHT, within the skin. However, our knowledge about the relationship between circulating DHT and its local action remains limited. Usually, we expect direct correlation between hormone concentration in blood and its presence near/inside the target cells, a classic endocrine paradigm, which holds true in most circumstances. On the other hand, especially with regard to androgens’ action, paracrine and intracrine mechanisms should also be considered [42]. The example of prostate provides evidence of possible discrepancies between serum DHT levels and its intracrine production, and in consequence, the final DHT concentration within the gland. A similar situation is likely for pilosebaceous skin units. Excess DHT was found in our case; however, it does not mean that it was produced exclusively within the adrenals. Perhaps, progestin precursors were indeed of adrenal origin, while final steps of “backdoor” DHT biosynthesis took place in peripheral tissues, e.g., in the liver, fat tissue or both [43]. Furthermore, high circulating concentrations of DHT may potentially interfere with the hypothalamic-pituitary region and affect the natural rhythm for gonadotropin release. This could explain menstrual irregularities reported in the studied adolescent. 

### 4.9. Diagnostic Strategy

Non-classic congenital adrenal hyperplasia may present with a wide spectrum of clinical manifestations from early adrenarche/pubarche in children with mild bone age acceleration up to secondary true precocious or early puberty with progressive hyperandrogenism through the adolescent period [44,45,46,47]. Symptoms of androgen excess are obviously easier to notice in affected girls than in boys. However, in adolescent girls there are frequent diagnostic dilemmas while trying to distinguish between hyperandrogenism due to the late-onset non-classic disorder of adrenal steroidogenesis, mild innate alterations in peripheral steroid metabolism, or early progression of PCOS, and sometimes a combination of these problems [22]. In all these conditions normal cortisol levels are maintained and no changes in electrolyte balance are usually detectable, although a tendency for slightly or temporarily elevated ACTH may be observed. 

In the past, the diagnosis of CAH was routinely established based upon the analysis of serum steroid levels, focused specifically on 17OHP and androgens in relation to cortisol in response to ACTH_1–24_ stimulation test. A moderate raise of 17OHP used to be considered as confirmation for non-classic congenital adrenal hyperplasia [46]. Nevertheless, biochemical analyses relying either on blood tests or 24 h urine collections do not always allow drawing indubitable conclusions [48,49,50]. Interpretation of the urinary steroid profiles is complex and requires lots of experience. The collaborating laboratories are sometimes able to exclude CAH but do not provide any alternative diagnosis. Therefore, molecular analysis of the 21α-hydroxylase gene, *CYP21A2*, seems the most sensible approach. However, the gene located on chromosome 6p21.3 is situated only 30 kb from a pseudogene, *CYP21A1P*, which shares 98% sequence similarity with *CYP21A2* [51]. This feature renders the mutation analysis technically difficult, thus many laboratories use standardized predesigned multiplex ligation-dependent probe amplification (MLPA) assay, i.e., a panel of polymerase chain reaction (PCR) -based tests, which detect most large gene rearrangements (such as *CYP21A2* deletions, *21A2/21A1P* conversions) and several frequent gene mutations leading to serious impairment of enzymatic activity [52]. However, this method does not cover the variants typically responsible for non-classic CAH, e.g., P30L, V281L and P453S, associated with persisting 20–50% activity of 21α-hydroxylase. Their detection requires expensive sequence analysis, which is rarely performed in routine clinical diagnostics of non-classic CAH. Nonetheless, in some instances, molecular analysis would be helpful to differentiate between CAH and PCOS [53]. Moreover, the risk of transmission of CAH to the next generation may be worth an extended genetic evaluation too. Accordingly, the option of genetic investigations and counseling was explained to the girl and her parents. 

Overall, our experience with hyperandrogenism in childhood indicates a necessity for combined analysis of its potential adrenal and gonadal origin, preferably at the earliest age possible, when hormonal function of gonads does not yet interfere with diagnosis of precocious, exacerbated adrenarche, or both. Investigations should consider the timing of symptoms together with the results of blood testing, including stimulation as well as suppression exams of the adrenal function, and also relate them to the urine steroid metabolites excretion. The separate analyses are frequently not fully informative. In-depth investigations and searching for androgen precursors and metabolites in blood tests and urines seems to be a practical approach when an alternative pathway is taken into account. The evaluation of DHT level and confirmation of adrenal contribution in DHT synthesis is also of cardinal importance.

### 4.10. Treatment Options

The general therapeutic aim in congenital adrenal hyperplasia is to supplement hormone deficiencies and to suppress excess synthesis of adrenal androgens. The pharmacological treatment of classic CAH relies upon chronic use of oral glucocorticoids. Hydrocortisone is conventionally considered as the first choice because its molecule is identical to endogenous cortisol; however, synthetic steroids such as prednisone or dexamethasone are also used in adult patients [7,9,10,13]. New therapeutic modalities based on modified release of hydrocortisone, capable of mimicking the normal diurnal profile of cortisol secretion and reducing early-morning ACTH surge and subsequent androgen production, have recently been developed [54]. Moreover, it appears that delayed-release hydrocortisone preparation exerts much improved control of alternative pathway-mediated androgen excess in patients suffering from 21α-hydroxylase deficiency [13]. Unfortunately, these modern medications are not always available due to elevated costs. The therapy of salt-losing CAH should be enriched with mineralocorticoid receptor agonist, 9α-fludrocortisone. In special situations (e.g., infection, injury, surgery and general anesthesia) the doses of glucocorticoids should be increased immediately, even 2–4 times, to prevent acute adrenal insufficiency (adrenal crisis). Adequate therapy determines the normal growth and development of the child. On the other hand, serious adverse effects, including iatrogenic Cushing’s syndrome, may emerge if long-term steroid replacement becomes supraphysiological. For that reason permanent medical care and regular assessment of growth velocity, body weight and blood pressure, as well as biochemical control are recommended. In fully symptomatic cases of classic CAH, girls with masculinization of the external genitalia additionally need surgical intervention, optimally in their infancy, with plausible re-evaluation later on, after puberty [7]. Despite the rigorous therapeutic regime for the classic forms of CAH, patients with non-classic CAH and moderate hyperandrogenism are usually not treated at all, although potential risk of adrenal insufficiency in the acute stress may persist. In practice, the patients are notified that symptoms are inherent features of their individual appearance without major effects on their health. In females displaying menstrual irregularity, there is a tendency to over-diagnose the polycystic ovary syndrome and to introduce the typical therapeutic scheme for this disease. However, the coexistence of both disorders, i.e., non-classic CAH inducing androgen “backdoor” pathway in adrenals and PCOS with activation of the same (“backdoor”) androgen steroidogenic pathway in ovaries should also be considered.

According to our investigations, the current case presents a very mild 21α-hydroxylase deficiency (or heterozygous status) with consequent enhancement of the androgen “backdoor” pathway in adrenals, peripheral tissues or both, using adrenal origin precursors. Eventually, considering the patient’s age, further control by an endocrinologist-gynecologist was recommended with suggested introduction of biphasic (estradiol/drospirenone) contraceptive pill to block the ovarian source of androgens, with concurrent low-dose spironolactone. Adrenal suppression (by glucocorticoid drugs) would be considered only in case of a lack of the anti-androgenic effects of the above-mentioned therapy or in the event of an exacerbation of symptoms. The patient was also given recommendations for dermatological care to control hirsutism and advice on healthy diet and regular physical activity to avoid the development of insulin resistance. 

## 5. Final Conclusions

We believe that diagnosis of PCOS in adolescent girls and young women should be established very carefully, taking into account innate disorders of adrenal steroidogenesis as a coexisting parallel problem, or as the main disease. The diagnostic procedure is not straightforward, usually takes time and requires several stages to collect data from basal analyses and functional tests, with estimation of serum steroids and their urinary metabolites. However, even evident clinical symptoms are not always in direct correlation with biochemical findings. Even mild limitation of 21α-hydroxylase activity in course of non-classic CAH is sufficient to shift adrenal androgen steroidogenesis toward the alternative “backdoor” pathway, which can be also be achieved outside the adrenal cortex. Therefore, paracrine and intracrine processes leading to active androgen biosynthesis in peripheral and target tissues should be considered in diagnosis. The analysis of urinary steroid metabolites is helpful because it reflects the status of several pathways of steroidogenesis as well as steroid peripheral metabolism by a single cross-sectional study. 

Taken together, it is essential to emphasize that diagnostics of hyperandrogenism in girls/women cannot be limited to crude estimation of total or free testosterone levels, which can be normal. Circulating DHT levels and also the androgen backdoor pathway of its synthesis should be considered equally. This approach may provide more precise data as steroid metabolites might suggest not only confirmation or exclusion of CAH, but sometimes also explain more complex steroidogenic and metabolic traces.

## Figures and Tables

**Table 1:** Results of basic hormonal serum estimations.

Hormones	Value	Reference Values
LH ^1^, (mIU/mL)	9.80	1.80–11.78
FSH ^2^, (mIU/mL)	6.30	3.00–8.10
E2 ^3^, (pg/mL)	38.00	21.00–251.00
ACTH ^4^, (pg/mL)	84.90	10.00–60.00
T ^5^, (nmol/L)	2.26	0.38–2.74
DHEA-S ^6^, (μmol/L)	11.39	3.17–14.39

^1^ LH luteinizing hormone; ^2^ FSH follicle stimulating hormone; ^3^ E2 estradiol; ^4^ ACTH adrenocorticotropic hormone; ^5^ T testosterone and ^6^ DHEA-S dehydroepiandrosterone sulfate.

**Table 2:** Results of the oral glucose tolerance test (75 g glucose load).

Time (min)	Glucose (mg/dL)	Insulin (μU/mL)	C-Peptide (pmol/mL)
0′	84	7.60	0.59
30′	160	45.50	-
60′	146	53.80	3.57
90′	111	45.70	-
120′	107	38.50	3.02
150′	111	32.70	-

**Table 3:** Results of intravenous stimulation test with Synacthen.

Time (min)	F ^1^ (ng/mL)	17OHP ^2^ (ng/mL)	ANDR ^3^ (ng/mL)
0′	212.00	1.66	3.00
60′	272.00	2.29	3.10
120′	261.00	2.38	3.59

^1^ F cortisol; ^2^ 17OHP 17α-hydroxyprogesterone; ^3^ ANDR androstenedione.

**Table 4:** Steroid profile in 24-h urine collection (gas chromatography mass spectrometry, GC-MS).

Steroid Metabolites	Results (μg/24 h)	Reference Range Values
AN (androsterone)	2102.5	(710–3140)
ET (etiocholanolone)	1994.7	(380–2590)
11-OAN/ET (11-ketoandrosterone/etiocholanolone)	103.5	
11-OHAN (11-hydroxy-androsterone)	271.6	(180–912)
11-OHET (11-hydroxy-etiocholanolone)	55.0	(54–750)
ET/AN	0.9	
DHEA (dehydroepiandrosterone)	438.2	(73–559)
5-AND (5-androstenediol)	131.5	(16–180)
**16α-OHDHEA (16alpha-hydroxy-DHEA)**	**610.2**	**(50–490)**
An-3-ol (5-androstenetriol)	357.5	(130–610)
5-PT (5-pregnenetriol)	175.9	(80–390)
16-OHPN (16alpha-hydroxy-pregnenolone)	0.0	
17-OHPN (5beta) (17-hydroxy-pregnanolone)	124.9	(25–208)
17-OHPN (5alpha) (17-hydroxy-pregnanolone)	6.1	(0.5–9)
PT (pregnanetriol)	385.0	(185–885)
**PTN (pregnanetriolone)**	**142.5**	**(10–50)**
PD (pregnanediol)	227.2	<900
E1 (estrone)	3.7	(0–52)
E2 (estradiol)	1.4	(0–15)
E3 (estriol)	4.0	(1–30)
THS (tetrahydro-11-deoxycortisol)	36.2	(20–72)
THDOC (tetrahydro-11-deoxycorticosterone)	4.9	(<16)
THA (tetrahydro-11-dehydrocorticosterone)	83.2	(50–260)
allo-THA (allo-tetrahydro-11-dehydrocorticosterone)	24.6	
THB (tetrahydro-corticosterone)	66.2	(20–256)
allo-THB (allo-tetrahydro-corticosterone)	79.0	(78–543)
THAldo (tetrahydro-aldosterone)	25.6	(7–51)
THE (tetrahydro-cortisone)	1995.6	(585–3960)
THF (tetrahydro-cortisol)	771.4	(315–2060)
allo-THF (allo-tetrahydro-cortisol)	425.0	(420–2660)
THF/allo-THF	2.2	
THF+allo-THF/THF	0.6	(0.55–1.2)
a-CTN (alpha-cortolone)	1065.8	(360–1420)
b-CTN (beta-cortolone)	273.8	(75–925)
a-CT (alpha-cortol)	154.7	(30–800)
b-CT (beta-cortol)	110.7	(60–300)
E (cortisone)	70.3	(17–115)
F (cortisol)	46.8	(9–52)
F/E (cortisol/cortisone)	0.7	(0.35–0.75)
6b-OHF (6beta-hydrocortisol)	0.0	
20a-DHF (20alfa/beta-dihydrocortisol)	0.0	

In the table, bold was used to emphasize that both compounds do not fall within the reference range values, which is clinically relevant.

**Table 5:** Results of the three-day dexamethasone suppression test.

	ACTH ^1^ (pg/mL)	DHEA-S ^2^ (μmol/L)	ANDR ^3^ (ng/mL)	T^4^ (nmol/L)	DHT ^5^ (pg/mL)	F ^6^ (ng/mL)
baseline reference	10.00–60.00	3.17–14.39	0.14–2.92	0.38–2.74	24.00–368.00	37.00–194.00
values baseline	37.20	16.40	2.59	3.84	578.00	203.00
post-test	0.90	4.52	1.78	2.16	337.00	<10.00

^1^ ACTH adrenocorticotropic hormone; ^2^ DHEA-S dehydroepiandrosterone sulfate; ^3^ ANDR androstenedione; ^4^ T testosterone; ^5^ DHT dihydrotestosterone; ^6^ F cortisol.
